# Edaravone alleviates cell apoptosis and mitochondrial injury in ischemia–reperfusion-induced kidney injury via the JAK/STAT pathway

**DOI:** 10.1186/s40659-020-00297-0

**Published:** 2020-07-03

**Authors:** Xiaoying Zhao, Erfei Zhang, Xiaofen Ren, Xiaoli Bai, Dongming Wang, Ling Bai, Danlei Luo, Zheng Guo, Qiang Wang, Jianxin Yang

**Affiliations:** 1 Department of Anesthesiology, Second Hospital of Shanxi Medical University, Taiyuan, China; 2 Department of Anesthesiology, The Affiliated Hospital of Yan’an University, Yan’an, China; 3 Xi’an Jiaotong University Health Science Center, Xi’an, China; 4 The First Affiliated Hospital of Xi’an Jiaotong University, Xi’an, China

**Keywords:** Ischemia–reperfusion injury, Edaravone, JAK/STAT, Mitochondria

## Abstract

**Background:**

Kidney ischemia–reperfusion injury is a common pathophysiological phenomenon in the clinic. A large number of studies have found that the tyrosine protein kinase/signal transducer and activator of transcription (JAK/STAT) pathway is involved in the development of a variety of kidney diseases and renal protection associated with multiple drugs. Edaravone (EDA) is an effective free radical scavenger that has been used clinically for the treatment of postischemic neuronal injury. This study aimed to identify whether EDA improved kidney function in rats with ischemia–reperfusion injury by regulating the JAK/STAT pathway and clarify the underlying mechanism.

**Methods:**

Histomorphological analysis was used to assess pathological kidney injury, and mitochondrial damage was observed by transmission electron microscopy. Terminal deoxynucleotidyl transferase-mediated dUTP nick end-labeling (TUNEL) staining was performed to detect tubular epithelial cell apoptosis. The expression of JAK2, P-JAK2, STAT3, P-STAT3, STAT1, P-STAT1, BAX and Bcl-2 was assessed by western blotting. Mitochondrial function in the kidney was assessed by mitochondrial membrane potential (ΔΨm) measurement.

**Results:**

The results showed that EDA inhibited the expression of p-JAK2, p-STAT3 and p-STAT1, accompanied by downregulation of the expression of Bax and caspase-3, and significantly ameliorated kidney damage caused by ischemia–reperfusion injury (IRI). Furthermore, the JC-1 dye assay showed that edaravone attenuated ischemia–reperfusion-induced loss of kidney ΔΨm.

**Conclusion:**

Our findings indicate that EDA protects against kidney damage caused by ischemia–reperfusion through JAK/STAT signaling, inhibiting apoptosis and improving mitochondrial injury.

## Background

Ischemia–reperfusion injury (IRI) is a common pathophysiological phenomenon and is one of the momentous causes of acute renal failure (ARF) [1]. Clinically, shock, sepsis, cardiac arrest, nephrectomy, kidney transplantation and other complicated kidney surgery may cause kidney IRI [2, 3]. Therefore, effective treatment to quickly reduce injury after kidney ischemia–reperfusion (IR) is urgently needed.

Studies have shown that the production of reactive oxygen species (ROS) is closely related to IRI. ROS are derived from molecular oxygen and are formed by redox reactions or electronic excitation. ROS can be divided into nonradical and free radical (with at least one free electron) species. Hydrogen peroxide (H_2_O_2_) and O_2_**·−** are recognized as the major ROS associated with redox regulation of biological activities. Increased ROS causes cell apoptosis in organs with ischemia–reperfusion injury, including the kidney [4]. Excessive ROS generation leads to alterations in the posttranscriptional modification of a large number of proteases, resulting in an imbalance in the biological activity of these proteases, which causes pathological conditions in organs [5]. Studies suggest that signaling by pattern recognition receptors (PRRs), such as Toll-like receptor 2 (TLR2) and the nucleotide-binding oligomerization domain-like receptors (NLRs) NOD1 and NOD2, induces neutrophil infiltration into ischemic kidneys following ischemic insult; TLR2 or NOD1/2 blockade could decrease neutrophil-mediated inflammation, which alleviates renal ischemia–reperfusion injury [6]. A study also indicated that TNF-α sustains neutrophil recruitment during inflammation through endothelial activation [7], and IL-6 regulates neutrophil trafficking during acute inflammation via STAT3 [8]. In addition, a study suggested that the release of many inflammatory factors, including TNF-α, IL-6, IFN-γ, etc., promotes renal tubular epithelial cell apoptosis through the Ras/MAPK pathway [9]. Therefore, reducing the release of inflammatory factors is very important in improving renal ischemia–reperfusion injury. Coincidentally, the anti-radical and anti-inflammatory effects of EDA have been reported [10]. EDA, an effective free radical scavenger, was first used clinically to treat neuronal damage after acute cerebral ischemic stroke in 2001 [11], and to date, EDA has been used as a standard therapy for acute ischemic stroke in Japan. Many studies have shown that EDA has protective effects against IRI of organs, such as reducing multiple erosions and bleeding injury in the small intestine induced by clamping the superior mesenteric artery for 30 min. EDA (10 mg/kg) was delivered intravenously before ischemia and after reperfusion to prevent neutrophil activation and reduce renal injury. EDA was administered into the femoral artery after reperfusion for 30 min, reducing malondialdehyde levels and preventing lipid peroxidation-mediated ischemia–reperfusion injury of the bladder. EDA reduced myeloperoxidase and malondialdehyde levels, indicating that EDA reduces oxidative stress and prevents testicular damage induced by ischemia–reperfusion [12–15].

The JAK/STAT pathway is an important member of a group of intracellular signal transduction pathways that were discovered in recent years and participates in cellular apoptosis, inflammatory responses and oxidative stress reactions. Previous studies have demonstrated that inhibition of JAK/STAT pathway activation decreases ATP production, increases ROS levels, and increases cell death in IRI [16]. Studies have shown that animals treated with either dexmedetomidine or AG490 exhibited improved renal functional recovery, attenuated histological lesions and reduced numbers of apoptotic tubular epithelial cells due to inhibition of the JAK/STAT pathway in kidney IRI [17]. Ellagic acid exerted a renoprotective effect by suppressing the NOX4/JAK/STAT signaling pathway (ellagic acid suppressed the phosphorylation of JAK1, JAK2, and STAT1 and reduced the level of NOX4) and reduced apoptosis, hypoxia-induced inflammatory response, and ROS levels, resulting in reduced acute kidney injury in the clinic [18]. The tyrosine protein kinase 2 (Jak2)-specific inhibitor AG490 decreased Stat1 and Stat3 phosphorylation and promoted human proximal tubular epithelial cell apoptosis induced by ATP depletion/recovery [19]. Furthermore, the JAK/STAT pathway is involved in the pharmacological mechanisms of many drugs [17, 20, 21] that treat many kidney diseases, such as obstructive nephropathy [22], diabetic nephropathy [23, 24], and various types of glomerulonephritis [25, 26]. Therefore, inhibition of the JAK/STAT pathway is a viable strategy in the treatment of renal injury.

Mitochondria not only play a vital role in energy production in the normal physiological state but are also critical for the metabolism of ribose, lipids and proteins necessary for cellular survival. Prolonged hypoxia and other external stimuli lead to mitochondrial dysfunction, ATP exhaustion, calcium imbalance, pathological mitochondrial pore formation, and the release of apoptotic proteins, resulting in apoptosis [27, 28]. Increasing evidence has shown that STAT3 exerts nontranscriptional activity in mitochondria, regulating mitochondrial bioenergy through complexes I, II, and V in the electron transport chain [29–31]. STAT3 may also inhibit the formation of the mitochondrial permeability transition pore by interacting with cyclophilin D [32], thereby stabilizing the mitochondrial membrane potential (ΔΨm) required for biological functions and inhibiting the release of proteins and cytokines that may cause apoptosis [33]. Another study [20] showed that integrin-FAK inhibition reduced pS727-STAT3 within mitochondria and reduced mitochondrial function in a nontranscriptional manner, resulting in reduced phosphorylation of STAT3 in mitochondria and increased ΔΨm loss and ROS production. These data suggest that integrin-FAK activation promotes STAT3-dependent mitochondrial function and cell survival, which further suggests a new treatment strategy for cell survival using S727-STAT3 activators.

Consequently, it is reasonable to hypothesize that the protective effect of EDA on kidney IRI may be related to inhibition of the JAK/STAT pathway.

## Materials and method

### Animals

Male Sprague–Dawley rats (weighing 220–270 g) (provided by the Experimental Animal Center of the Academy of Military Sciences of the Chinese People’s Liberation Army) were housed under controlled conditions in a constant-humidity breeding house with a natural light/dark cycle, allowed free access to food and water, and randomly assigned into different groups. The experimental protocol was approved by the Ethics Committee for Animal Experimentation of the Second Hospital of Shanxi Medical (Project Approval No. 201706). All experiments were performed according to the Guidelines for Animal Experimentation of Shanxi Medical University (Taiyuan, Shanxi Province, China).

### Animal model

All rats were surgically prepared for kidney IR as previously described [34]. Briefly, after intraperitoneal anesthetization with 10% chloral hydrate (350 mg/kg), the kidneys of the animals were exposed via incisions (approximately 1.5 cm) along both sides of the spine at the lower edge of the costal. The bilateral kidneys were gently dissociated, and connective tissue such as fat was removed from around the kidney pedicle. Subsequently, noninvasive arterial clamps were placed on the bilateral kidney pedicles. Ischemia was confirmed by discoloration of the kidneys from bright red to dark red. After 45 min, successful reperfusion was obtained by removing the clamps, restoring the blood supply, which was accompanied by the color of the kidneys changing from dark red to bright red. The surgical incisions were stitched and covered with sterile gauze. After recovery from anesthesia, the rats were returned to their cages, remaining for 24 h in clean cages with free access to food and water. For the sham group, an identical operation was performed without clamping the bilateral kidney pedicles.

### Treatment protocol

The animals (n = 10/group) were treated with edaravone (3 mg/kg, 5 min before reperfusion, tail vein injection). A study showed that AG490, a specific antagonist of JAK2, significantly alleviated AKI through suppression of the JAK2/STAT3 pathway, reducing oxidative stress and inflammation [35]. Thus, we used AG490 as a positive treatment for AKI in our experiment, and 10 mg/kg AG490 was intraperitoneally injected 30 min before ischemia. The rats in the sham and IR groups were injected with the same amount of saline at the same time point. Blood and kidney samples were obtained at 24 h after kidney reperfusion. Blood samples were centrifuged at 3000 rpm for 20 min to obtain separated serum. The levels of serum urea nitrogen (BUN) and creatinine (Cr) were measured by an automatic biochemical analyzer. The right kidney tissue was frozen rapidly in liquid nitrogen and stored at − 80 °C, and the left kidney tissue was fixed in 4% paraformaldehyde for further analysis.

### Histomorphological analysis

The tissues that were collected in 4% paraformaldehyde were embedded in paraffin, sliced into 4 μm thick sections and subsequently stained with hematoxylin and eosin (HE). Kidney morphological changes were observed under a light microscope (×400). Twenty nonoverlapping fields were randomly chosen to determine kidney injury. Histopathological changes that were evaluated in our study included swelling, vacuolization, necrosis, desquamation, loss of brush border, cast formation and tubular dilation. Moreover, semiquantitative assessment of the histological lesions based on tubular necrosis was graded as follows: no damage; slight damage: lesion areas < 25%; moderate damage: lesion areas 25–50%; severe damage: lesion areas 50–75%; and the most serious damage: lesion areas > 75% [36].

### Transmission electron microscopy

Approximately 1 mm^3^ of fresh kidney tissue from the right kidney was immediately placed in 2.5% glutaraldehyde at 4 °C for 2 h and then fixed in 1.5% osmium tetraoxide. Serial dehydration was performed in acetone after fixation, followed by embedding in epoxy resin. Ultrathin sections were cut with an ultramicrotome and then stained with uranyl acetate and lead citrate. Transmission electron microscopy was used to observe ultrastructure of the mitochondria, and semiquantitative analysis was performed according to the Flameng classification method to determined mitochondrial damage.

### TUNEL staining

The TUNEL method was used to detect apoptotic nuclei with an apoptosis detection kit according to the manufacturer’s instructions. At least 20 random nonoverlapping fields were viewed with a light microscope (×400) and scored for the number of apoptotic nuclei (green) and total nuclei (blue). The apoptosis index (AI) of kidney tubular epithelial cells was calculated (AI = apoptotic nuclei/total nuclei × 100%).

### Mitochondrial membrane potential (ΔΨm) detection

Mitochondria were prepared from approximately 80 mg of fresh kidney cortex tissue. According to the protocol of the mitochondria isolation kit, 10 times ice-cold buffer A was added to the tissue, which was homogenized on ice. The isolated mitochondria were suspended in mitochondrial storage buffer to produce a suspension containing 10–20 mg/ml mitochondrial protein. JC-1, a lipophilic cationic dye that is highly specific for the mitochondrial membrane, was used to assess the ΔΨm. The red and green fluorescence intensities were detected by fluorescent enzyme markers, and the ratio of red/green fluorescence indicated the mitochondrial membrane potential level.

### Western blotting

Frozen kidney samples were homogenized on ice in RIPA lysis buffer containing standard protease and phosphatase inhibitors and centrifuged at 12,000 rpm and 4 °C for 5 min. The supernatant was collected, and the protein concentration was determined using a BCA assay kit. The proteins were separated by SDS-PAGE using 10% gels and then transferred to nitrocellulose membranes, which were blotted and probed overnight at 4 °C with the following antibodies: anti-JAK2 (SAB4501600, Sigma-Aldrich), anti-phospho-JAK2 (Tyr1007/1008, 07-606, Sigma-Aldrich), anti-STAT3 (SAB2104912, Sigma-Aldrich), anti-phospho-STAT3 (pTyr705, SAB4300033, Sigma-Aldrich), anti-STAT1 (AV38933, Sigma-Aldrich), anti- phospho-STAT1 (pTyr701, S2565-1VL, Sigma-Aldrich), anti- BAX (SAB3500343, Sigma-Aldrich), anti-Bcl-2 (SAB4500003, Sigma-Aldrich), anti-caspase-3 (C9598, Sigma-Aldrich) or anti-GAPDH (ab9485, Abcam). After the membranes were washed three times in TBST and incubated in HRP-conjugated secondary antibody for 1 h at room temperature, ECL luminescence solution was used to visualize the immunoreactive protein bands, and the bands were quantified using the Quantity One software package.

### Statistical analysis

GraphPad Prism 6.0 was used to perform all statistical analyses. All values are presented as the mean ± standard error of the mean (SEM) and were analyzed by one-way analysis of variance (ANOVA). If the ANOVA result was significant, then the data were analyzed by pairwise multiple comparison procedures using Tukey’s multiple comparison test. A type I error was defined as < 0.5, and the significance was set at a probability of *p* < 0.05.

## Results

### EDA improved IR-induced kidney injury

**Fig. 1:**
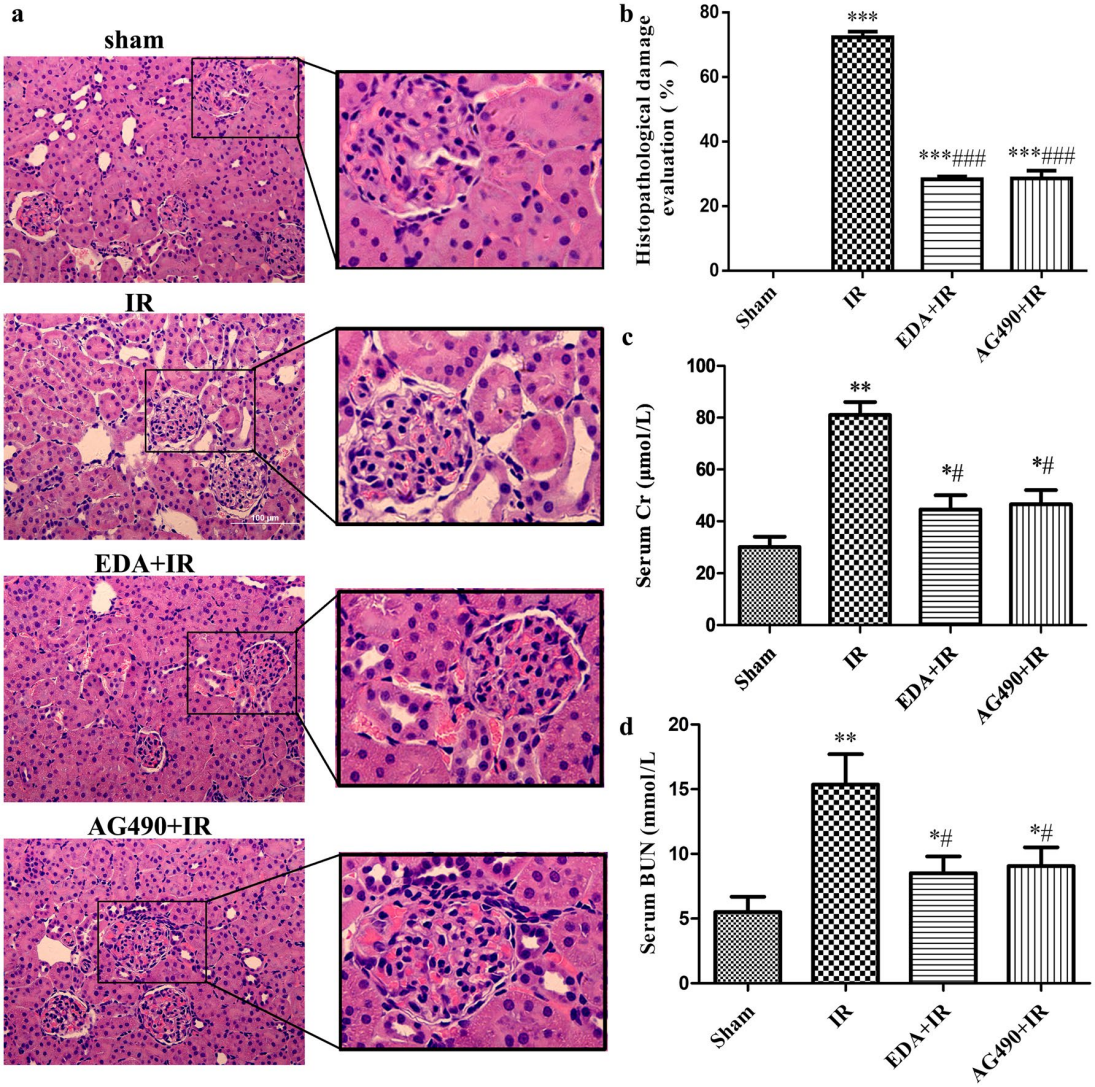
Edaravone improved IR-induced kidney injury. **a** Histopathological examination was performed using hematoxylin and eosin (HE), the typical representative glomerular and partial tubular structure was shown in the small box in the left image, and then we magnified the small box by 2.5× magnification as shown in right images. **b** The data is presented as the percentage of the damaged area in the total image under the light microscope referring to the (×400), bar graph showed the pathological damage percent of kidney injury compared with every other group. **c**, **d** The contents of serum urea nitrogen (BUN) and creatinine (Cr) were used to evaluate kidney function in each group. Data are represented as mean ± SEM (n = 10 per group) *P < 0.05, **P < 0.01, ***P < 0.001 vs. sham; ^#^*P *< 0.05, ^###^*P *< 0.001 vs. IR

To determine the effects of EDA on kidney IRI, HE staining was used to examine kidney injury. Many studies suggest that AG490 (a specific antagonist of JAK2) significantly prevents kidney injury [17, 35, 37]. Thus, we used AG490 as a positive treatment for kidney injury. The groups were divided into the sham, IR, EDA + IR and AG490 + IR groups, and each group was treated as described in the treatment protocol. Histological sections of kidney tissues from rats in the sham group showed normal glomerular and tubular architecture. In contrast, there were significant tubular changes following IR compared to that of sham-operation rats, as indicated by loss of brush borders, tubular dilation, cellular vacuolization, tubular cell swelling, degeneration and necrosis of kidney tubular epithelial cells and massive interstitial infiltration of inflammatory cells (Fig. 1a). Histopathological kidney damage was determined by calculating the percentage of damage in the total image (×400). The histopathological damage percentage was higher in the IR group than in the sham group (*p *< 0.01, Fig. 1b). However, the histopathological damage percentage was alleviated in the EDA + IR group (Fig. 1a). EDA significantly reduced the histopathological damage percentage in the EDA + IR group compared with the IR group (*p *< 0.05, Fig. 1b). Studies have shown that activation of the JAK/STAT signaling pathway contributes to the development of acute kidney injury [38, 39]; thus, we used the JAK2-specific antagonist AG490 as a positive control treatment of kidney injury. We found that pathological damage was also alleviated in the AG490 + IR group (Fig. 1a), and the histopathological damage percentage in the AG490 + IR group was lower than that in the IR group (*p *< 0.05, Fig. 1b). IR induced more macrophage and neutrophil infiltration into renal ischemia–reperfusion-injured tissues [40, 41]. The CD68 and MPO levels represent the infiltration of macrophages and neutrophils, respectively, in the tissue. Thus, we assessed the levels of CD68 and MPO in each group. The results indicated that IRI increased MPO and CD68 levels in IRI kidney tissue (*p *< 0.001, Additional file 1: Figure S1), but after EDA and AG490 treatment, MPO and CD68 levels were reduced in IRI kidney tissue (*p *< 0.001, Additional file 1: Figure S1). These results suggest that EDA and AG490 treatment alleviated macrophage and neutrophil infiltration into renal ischemia–reperfusion-injured tissues.

We further evaluated the levels of inflammatory factors in renal tissue after IR. The results indicated that IR increased TNF-α, IL-6, and IL-1β levels in the IR group compared with the sham group (*p *< 0.001, Additional file 2: Figure S2). However, EDA and AG490 treatment significantly reduced the TNF-α, IL-6, and IL-1β levels in the EDA + IR group and the AG490 + IR group (*p *< 0.001, Additional file 2: Figure S2).

We also measured the levels of Cr and BUN in serum to further assess renal function. As expected, the Cr and BUN levels in the IR group were higher than those in the sham group, indicating kidney dysfunction (*p *< 0.01, Fig. 1c, d). However, EDA and AG490 treatment significantly reduced the Cr and BUN levels in the EDA + IR group and the AG490 + IR group (*p *< 0.05, Fig. 1c, d). These results indicate that IR-induced kidney injury was alleviated by EDA, and the effects were the same as those of AG490. Thus, EDA may improve kidney injury by inhibiting JAK2.

### EDA inhibited kidney tubular epithelial cell apoptosis

**Fig. 2:**
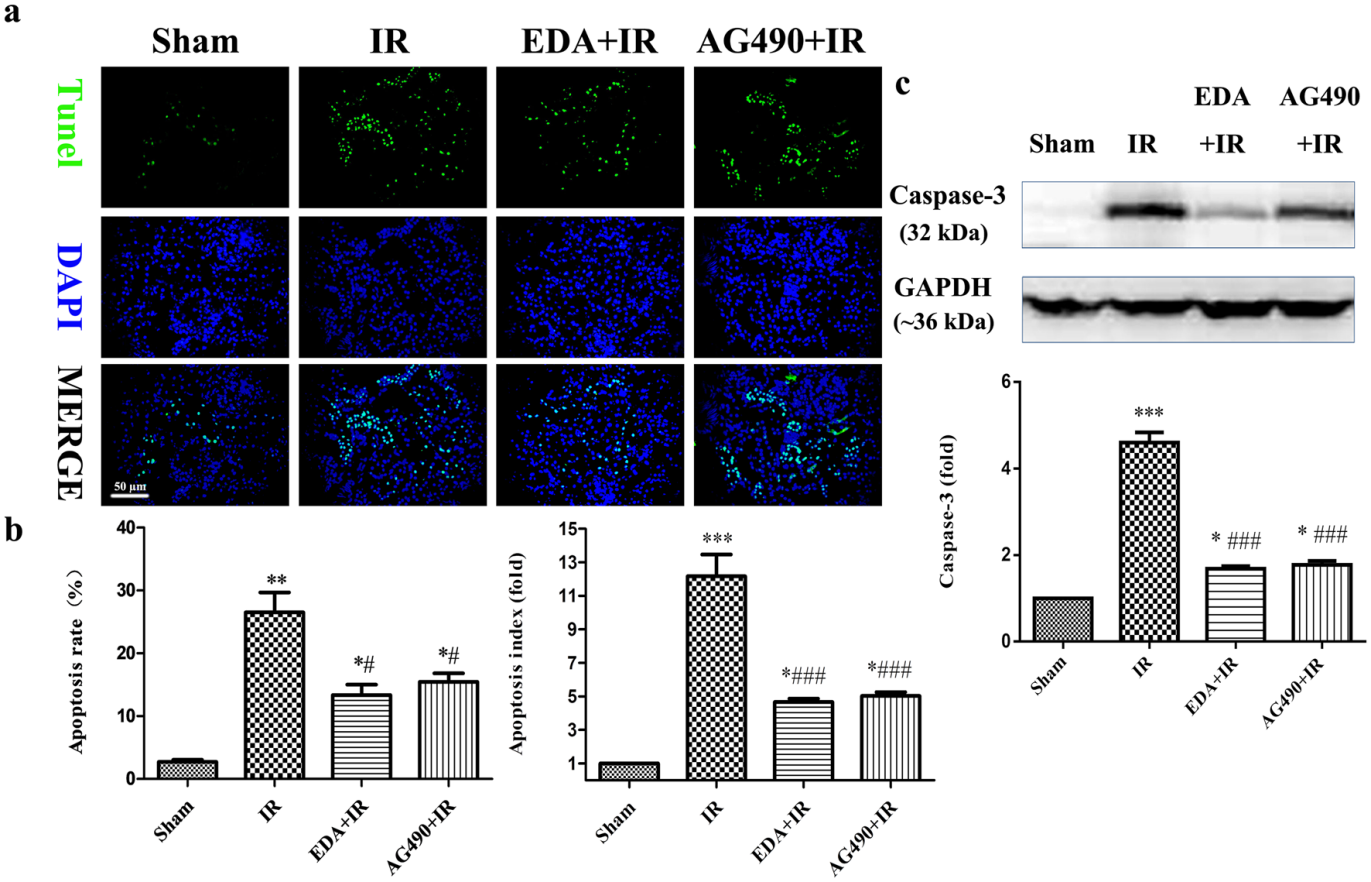
Edaravone inhibited IR-induced apoptosis in kidney tubular epithelial cells. **a** The terminal deoxynucleotidyl transferase-mediated dUTP nick end-labeling (TUNEL) was performed to detect the apoptotic nuclei. **b** The left bar graph showed the apoptosis rate in each group and the right bar graph showed the apoptosis rate normalized by the sham data in each group. **c** The up image is showed the changes of apoptotic proteins caspase-3 in each group were detected by western blot and the below bar graph showed the level of caspase-3 normalized by the sham data in each group. Data are represented as mean ± SEM (n = 10 per group) *P < 0.05, **P < 0.01, ***P < 0.001 vs. sham; ^#^*P *< 0.05, ^###^*P *< 0.001 vs. IR

The TUNEL assay was used to evaluate kidney IR-induced tubular epithelial cell apoptosis. A large number of apoptotic tubular epithelial cells were visible in the kidneys in the IR group, and the apoptosis rate of the IR group was higher than that of the sham group (*p *< 0.01, Fig. 2a, b). However, the apoptosis rates of tubular epithelial cells in the EDA + IR and AG490 +IR groups were lower than those in the IR group (*p *< 0.05, Fig. 2a, b). Moreover, western blot analysis showed that caspase-3 protein levels were higher in the IR group than in the sham group (*p *< 0.001, Fig. 2c), and caspase-3 protein levels were lower in the EDA + IR or AG490 + IR groups than in the IR group (*p *< 0.001, Fig. 2c). These results suggest that EDA treatment inhibited the apoptosis of kidney tubular epithelial cells after kidney IRI, and the effects were the same as those of AG490. Thus, EDA inhibited kidney tubular epithelial cell apoptosis possibly by inhibiting JAK2.

### EDA attenuated IR-induced kidney mitochondrial injury

**Fig. 3:**
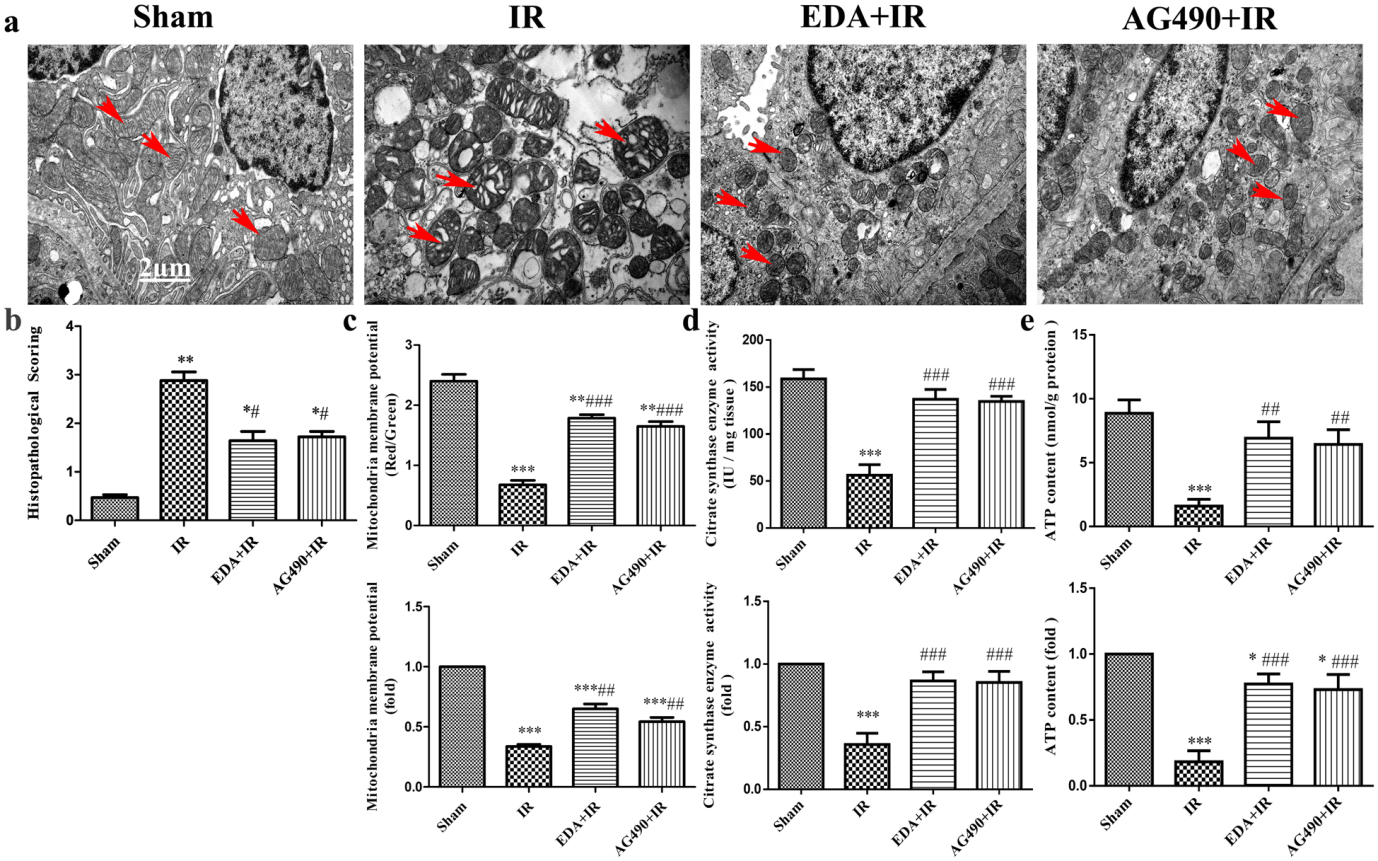
Edaravone attenuated IR-induced kidney mitochondrial injury. **a** Ultrastructural of mitochondria was analyzed by transmission electron microscopy (×20,000). **b** Flameng semi-quantitative assessment of the mitochondria ultrastructural based on mitochondrial damage. **c** JC-1 dye was used to detect the mitochondrial membrane potential (ΔΨm) in each group by fluorescence microplate reader. The up bar graph was the ratio of red/green fluorescence indicated the ΔΨm level and the below bar graph was the ΔΨm standardized by sham. **d**, **e** The mitochondrial function was evaluated by CS enzyme activity and ATP, the up bar graph was the level of CS enzyme activity and ATP in the each group, and the below bar graph was the level of CS enzyme activity and ATP normalized by the sham data in each group. Data are represented as mean ± SEM (n = 10 per group) *P < 0.05, **P < 0.01, ***P < 0.001 vs. sham; ^#^*P *< 0.05, ^##^*P *< 0.01, ^###^*P *< 0.001 vs. IR

Mitochondrial injury may result in cell death. To further determine the effect of EDA on mitochondria, ultrathin electron microscopy sections were prepared, and mitochondrial ultrastructures were observed with a transmission electron microscope. Marked injury, accompanied by mitochondrial cristae rupture, mitochondrial vacuolation and swelling, and matrix coagulation were observed in the IR group (the red arrows are shown), and these injuries were alleviated in both the EDA and AG490 treatment groups (Fig. 3a). The histopathological scores of kidney mitochondrial damage are presented in Fig. 3b. Compared with that of the sham group, the semiquantitative pathological score of kidney mitochondrial injury in the IR group was increased (*p *< 0.01, Fig. 3b) and was decreased significantly in the EDA and AG490 treatment groups (*p *< 0.05, Fig. 3b). Furthermore, the JC-1 dye assay was used to evaluate the ΔΨm. Compared with that of the sham group, the kidney ΔΨm was decreased in the IR group (*p *< 0.001, Fig. 3c upper bar graph), and this change was significantly prevented by pretreatment with EDA or AG490 (*p *< 0.001, Fig. 3c upper bar graph). The mitochondrial membrane potential (ΔΨm) was standardized to that of the sham group, and the kidney ΔΨm was lower in the IR group than in the sham group (*p *< 0.001, Fig. 3c lower bar graph); however, the kidney ΔΨm was increased in the EDA and AG490 treatment groups (*p *< 0.01, Fig. 3c lower bar graph). We further evaluated mitochondrial function by assessing ATP levels and CS enzymatic activity. The levels of CS enzymatic activity and ATP were lower in the IR group than in the sham group (*p *< 0.001, Fig. 3d upper bar graph). However, the levels of CS enzymatic activity and ATP were increased in the EDA and AG490 treatment groups (*p *< 0.001 and *p *< 0.01, respectively, Fig. 3d upper bar graph). The levels of CS enzymatic activity and ATP were normalized to that of the sham group, and the levels of CS enzymatic activity and ATP were lower in the IR group than in the sham group (*p *< 0.001, Fig. 3d lower bar graph); however, the levels of CS enzymatic activity and ATP were increased in the EDA and AG490 treatment groups (*p *< 0.001, Fig. 3d lower bar graph).

Improvements in mitochondrial function are beneficial in reducing the production of ROS. We assessed the levels of H_2_O_2_ and Mn-SOD. The levels of H_2_O_2_ and Mn-SOD were higher in the IR group than in the sham group (*p *< 0.001, Additional file 3: Figure S3a, b). However, the levels of H_2_O_2_ and Mn-SOD were reduced in the EDA and AG490 treatment groups (*p *< 0.01 and *p *< 0.001, respectively, Additional file 3: Figure S3a, b).

These results indicate that EDA attenuated IR-induced mitochondrial injury and improved mitochondrial function, and the effects were the same as those of AG490. Thus, EDA may improve mitochondrial injury by inhibiting JAK2.

### EDA prevents apoptosis via the JAK/STAT pathway in IRI-induced kidneys

**Fig. 4:**
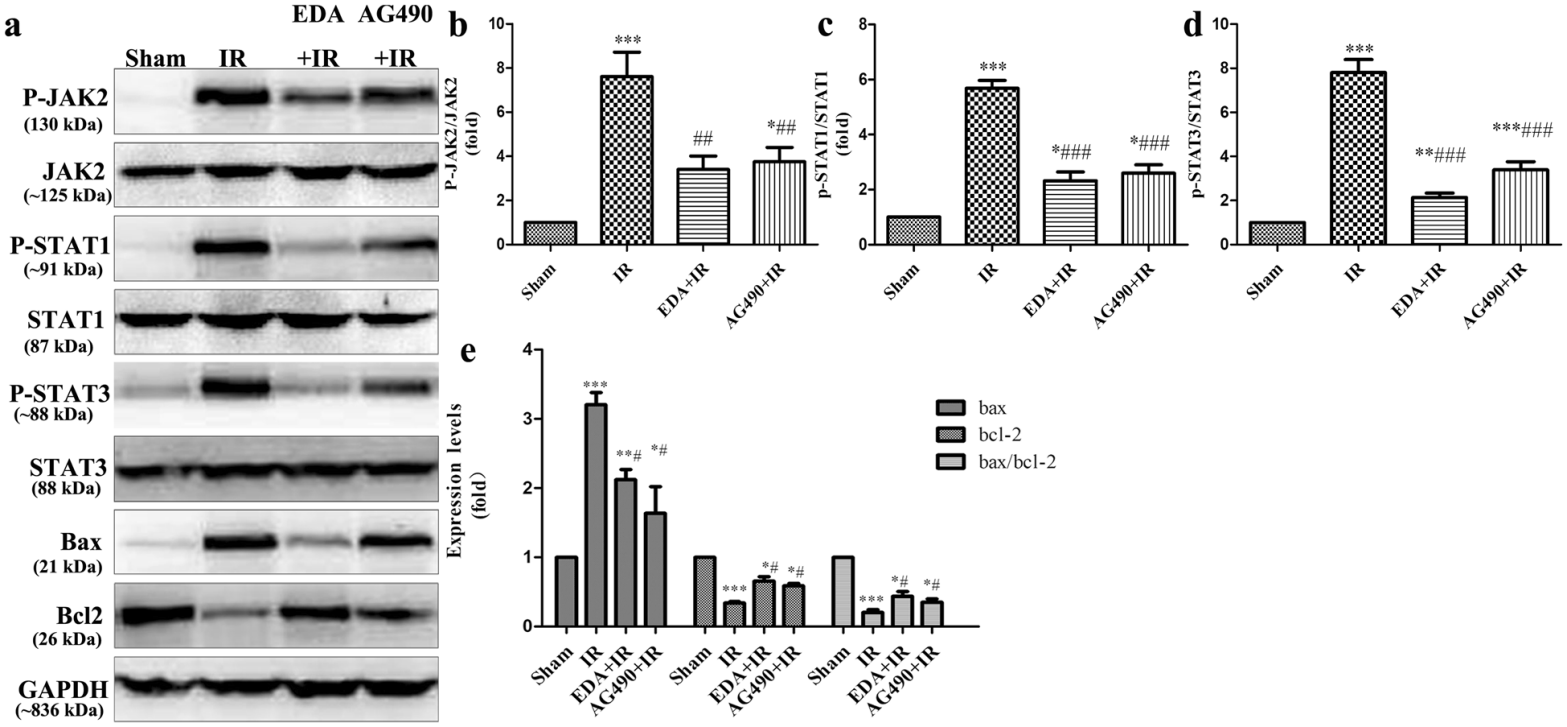
Edaravone inhibited IR-induced kidney apoptosis via JAK/STAT pathway. **a** Representative western blot bands showed that the expression of JAK2, p-JAK2, STAT1, p-STAT1, STAT3, p-STAT3, Bax and Bcl-2. **b**–**d** Bar graph showed the expression of p-JAK2/JAK2, p-STAT1/STAT1 and p-STAT3/STAT3 after normalized by the sham data. **e** Bar graph showed the expression of Bax, Bcl-2 protein and Bax/Bcl-2 after normalized by the sham data. Data are represented as mean ± SEM (n = 10 per group), *P < 0.05, **P < 0.01, ***P < 0.001 vs. sham; ^#^*P *< 0.05, ^##^*P *< 0.01, ^###^*P *< 0.001 vs. IR

To verify that EDA exerted renoprotective effects by inhibiting JAK/STAT signaling, we performed western blotting to analyze the phosphorylation of JAK2, STAT1 and STAT3. The expression of p-JAK2, p-STAT1 and p-STAT3 was weak in the sham group and was augmented in the IR groups, as shown in Fig. 4a. However, the expression of these three proteins was reduced in the EDA and AG490 groups (Fig. 4a). The data from each group were standardized to that of the sham group and then analyzed statistically. The results indicated that the expression of p-JAK2 protein was higher than total JAK2 in the IR group and the sham group (*p *< 0.001, Fig. 4b), but the p-JAK2 level was significantly reduced by EDA and AG490 treatment compared with that of IR group 2 (*p *< 0.01, Fig. 4b). Furthermore, the ratios of p-STAT1/STAT1 and p-STAT3/STAT3, the downstream molecules of the JAK2 cascade, were also higher in the IR groups than in the sham group (*p *< 0.001, Fig. 4c, d). The levels of p-STAT1 and p-STAT3 were reduced by EDA and AG490 treatment compared with those of the IR group (*p *< 0.001, Fig. 4c, d). These results suggest that EDA inhibited JAK2/STAT activation.

A previous study indicated that increased phosphorylation of JAK2 and STAT3 promotes cell apoptosis [42]. In addition, our results suggested that EDA prevented kidney tubular epithelial cell apoptosis, as shown in Fig. 3. Furthermore, we explored whether EDA exerted this protective effect on kidney IRI through the JAK/STAT pathway. We analyzed the antiapoptotic protein Bcl-2 and proapoptotic protein Bax in ischemic kidneys. The results showed that Bax and the Bax/Bcl-2 ratio were higher and Bcl-2 was lower in the IR group than in the sham group; however, Bax and the Bax/Bcl-2 ratio were lower and Bcl-2 was higher in the EDA treatment group than in the IR group (*p *< 0.05, Fig. 4e). We also found that Bax and the Bax/Bcl-2 ratio were reduced and Bcl-2 was increased after pretreatment with the antagonist of JAK2 (AG490), and the effects were the same as those of EDA treatment in kidney IRI. Taken together, these results indicated that EDA prevents apoptosis via the JAK/STAT pathway in IR-induced kidneys.

## Discussion

As a highly perfused organ, the kidney is particularly sensitive to IRI, which remains a major challenge in the treatment of many kidney diseases [3]. However, the pathophysiological mechanism of kidney IRI is very complicated and has not yet been fully elucidated, and the production of ROS stimulates tissue inflammation and induces NLRP3 inflammasome activation, resulting in cell death [4]. EDA is an effective free radical scavenger that has been clinically certified. Many laboratory studies and clinical trials have shown that EDA has a protective effect against various diseases in many different organs [13, 15, 43–46]. In the present study, we found that EDA reduced the levels of Cr and BUN, prevented kidney tubular epithelial cell apoptosis, and alleviated pathological injury to kidney tubules and kidney tubular epithelial cells in rats with IRI (Fig. 1). However, the underlying mechanism remains unknown.

Apoptosis of kidney tubular epithelial cells has become increasingly recognized as one of the mechanisms in IRI [47]. Among the multiple signal transduction pathways that initiate apoptosis in the body, the mitochondrial apoptosis pathway is the most important [28]. Moreover, Bcl-2 family proteins are capable of mediating the integrity of mitochondria [48, 49]. The relative levels of antiapoptotic and proapoptotic Bcl-2 family proteins, which regulate the mitochondrial apoptotic pathway, determine whether a cell will live or die [50]. Our results also indicated that IR induced kidney tubular epithelial cell apoptosis, as shown in Figs. 1 and 2. IR significantly induced mitochondrial damage, as shown by ROS accumulation, CS enzymatic activity and ATP reduction, as shown in Fig. 3. However, EDA significantly reduced mitochondrial damage, as indicated by reductions in ROS and CS enzymatic activity and improvements in ATP. EDA decreased Bax and increased Bcl-2 levels in kidney IRI (Fig. 4).

Numerous studies have indicated that the JAK/STAT pathway is involved in the mitochondrial apoptotic system, and both antiapoptotic and proapoptotic functions have been attributed to JAK/STAT activity in various signaling systems [32, 51, 52]. For example, Wen SH et al. found that inhibition of JAK/STAT can reduce IR-induced intestinal mucosal injury and apoptosis [53]; another study showed that inhibition of JAK2 phosphorylation inhibited downstream STAT1/STAT3 signaling and thus protected rat myocardial microvascular endothelial cells from anoxia and reoxygenation by reducing apoptosis-related protein expression and inhibiting apoptosis [51]. In addition, the JAK/STAT pathway is involved in the protective mechanisms of some drugs against kidney IRI [54]. In this study, we found that the phosphorylation of JAK2, STAT1 and STAT3 was upregulated by IR, accompanied by downregulation of Bcl-2 and upregulation of Bax and caspase-3. Treatment with EDA significantly inhibited the expression of the proapoptotic proteins Bax and caspase-3, promoted the expression of the antiapoptotic protein Bcl-2, and reduced the rate of apoptosis, ameliorating kidney function in rats. Some reviews have indicated that inflammatory factors (such as il-6, il-2, il-10, and il-3) phosphorylate STAT3 through JAK in inflammation and cell survival conditions, and IFN-γ can phosphorylate STAT1 through JAK2 and phosphorylate STAT3 through JAK1 to induce an inflammatory response and immunoregulation [55, 56]. Our results indicated that EDA significantly reduced il-6, TNF-α and il-1β levels (Additional file 2: Figure S2). EDA also significantly inhibited the phosphorylation of Stat1 and Stat3 (Fig. 4).

To further explore the relationship between the renoprotective effect of EDA and the JAK/STAT pathway, the JAK2-specific inhibitor AG490 was administered 30 min before ischemia. The results indicated that AG490 downregulated the phosphorylation of JAK2, STAT1 and STAT3, which was similar to the effect of EDA in our study. However, AG490 is a specific inhibitor of JAK2, and the JAK1 pathway is not inhibited by AG490. Thus, AG490 can partly inhibit the phosphorylation of Stat1 and Stat3. As shown in Fig. 4, we found that AG490 significantly reduced the level of p-JAK compared with that of IR, and the p-stat1 and p-stat2 levels were significantly lower than those in the IR group without AG490 treatment, but the p-stat1 and p-stat2 levels did not return to normal.

Since renal function depends on mitochondrial homeostasis, it has been suggested that mitochondrial alterations contribute to AKI development [57], and kidney ischemia–reperfusion (IR) causes mitochondrial injury and oxidative stress, as indicated by swollen and fragmented mitochondria with reduced membrane potential and increased generation of reactive oxygen species (ROS) and nitric oxide (NO) [58]. Mitochondrial dysfunction leads to apoptosis, and the loss of ΔΨm is a hallmark event of early apoptosis [5]. Our results suggested that EDA and AG490 significantly reduced mitochondrial damage, as indicated by improvements in ΔΨm, CS enzymatic activity, and ATP production and reduced ROS (H_2_O_2_) accumulation (Fig. 3). Various drugs can regulate mitochondrial function through the JAK/STAT pathway and thereby regulate cell survival or apoptosis [52, 59]. A large number of studies have indicated that mitochondrial STAT3 is responsible for mitochondrial damage during IR. Integrin-FAK-STAT3 signaling was involved in suppressing these cell death mechanisms by rapidly and potently promoting mitochondrial function, ΔΨm loss, and decreased ROS production, thereby boosting cell survival [20]. In summary, our current data showed that EDA can mitigate the loss of ΔΨm and mitochondrial microstructural damage by inhibiting the JAK/STAT pathway.

Although our results indicate that EDA significantly alleviated IR-induced kidney injury, in some experiments, EDA partly prevented the damage induced by reperfusion. We believe that IR induces kidney injury via very complex mechanisms and multiple regulatory pathways, such as necrocytosis, the inflammatory response, activation of the immune system, oxidative stress, mitochondrial injury, ROS-mediated mechanisms, the JAK/STAT pathway and IL-17C-mediated mechanisms [60–63]. Therefore, the damage is not completely inhibited by EDA, which exerts antioxidant effects and reduces mitochondrial damage. AG490 also had a specific inhibitory effect on JAK2 in our study. Therefore, there are other mechanisms and pathways playing damaging roles. Another issue is that EDA is a better alternative than AG490 for treating kidney IRI. A study suggested that inhibition of JAK2/STAT by AG490 can alleviate inflammation and regulate the function of immune cells [56]. However, EDA is an oxygen radical scavenger that can prevent ischemia–reperfusion injury in various tissues. A study also indicated that in addition to scavenging free radicals, edaravone has antiapoptotic, antinecrotic and anticytokine effects in cardiovascular diseases and stroke [64]. These antinecrotic, antiapoptotic and anticytokine effects of EDA are also involved in renoprotection. These effects of EDA are presented in Fig. 1 (decreased renal tubular epithelial cell necrosis), Fig. 2 (decreased cell apoptosis in renal tissue) and Additional file 2: Figure S2 (reduced production of inflammatory cytokines).

## Conclusion

Taken together, our findings suggest that the JAK/STAT pathway is at least partly involved in EDA-mediated suppression of cell death mechanisms in IR-induced kidney injury by increasing ΔΨm and maintaining mitochondrial function and integrity. These findings will provide new targets for EDA development and clinical treatment and provide a new direction for future research.

## Supplementary information

**Additional file 1: Figure S1.** Edaravone reduces CD68 and MPO level in IR-induced kidney injury (a) The up image was the representative western blot band showed that the expression of CD68. And the below bar graph showed the expression of CD68 normalized by the sham data in each group. (b) The contents of MPO were assessed. Data are represented as mean ± SEM (n = 10 per group), ^***^P < 0.001 vs. sham; ^###^*P *< 0.001 vs. IR.

**Additional file 2: Figure S2.** Edaravone reduces inflammatory factor production in IR-induced kidney injury. The inflammatory factors were assessed by ELISA, (a) TNF-α, (b) IL-6 and (c) IL-1β. Data are represented as mean ± SEM (n = 10 per group) ^*^P < 0.05, ^***^P < 0.001 vs. sham; ^###^*P *< 0.001 vs. IR.

**Additional file 3: Figure S3.** Edaravone reduces H_2_O_2_ and Mn-SOD level in IR-induced kidney injury. The H_2_O_2_ and Mn-SOD was assessed, (a) H_2_O_2_, (b) Mn-SOD. Data are represented as mean ± SEM (n = 10 per group), ^**^P < 0.01, ^***^P < 0.001 vs. sham; ^#^*P *< 0.05, ^##^*P *< 0.01, ^###^*P *< 0.001 vs. IR.

## Data Availability

This work was supported by the Health commission of Shanxi province (Grant No. 2018059).

## Abbreviations

IR: Ischemia–reperfusion
IRI: Ischemia–reperfusion injury
JAK/STAT: Tyrosine protein kinase/signal transducer and activator of transcription
EDA: Edaravone
ΔΨm: Mitochondrial membrane potential
ARF: Acute kidney failure
ROS: Reactive oxygen species
Cr: Creatinine
BUN: Urea nitrogen
HE: Hematoxylin and eosin
TUNEL: Terminal deoxynucleotidyl transferase-mediated dUTP nick end-labeling
AI: Apoptosis index
SEM: Standard error of the mean
ANOVA: One-way analysis of variance

## Availability of data and materials

All the data generated or analyzed during this study is available.
