# Supplementary figures and images for: Edaravone alleviates cell apoptosis and mitochondrial injury in ischemia–reperfusion-induced kidney injury via the JAK/STAT pathway
Source: Biol Res. 2020 Jul 3;53:28. doi: 10.1186/s40659-020-00297-0 (PMC7333427; doi:10.1186/s40659-020-00297-0)

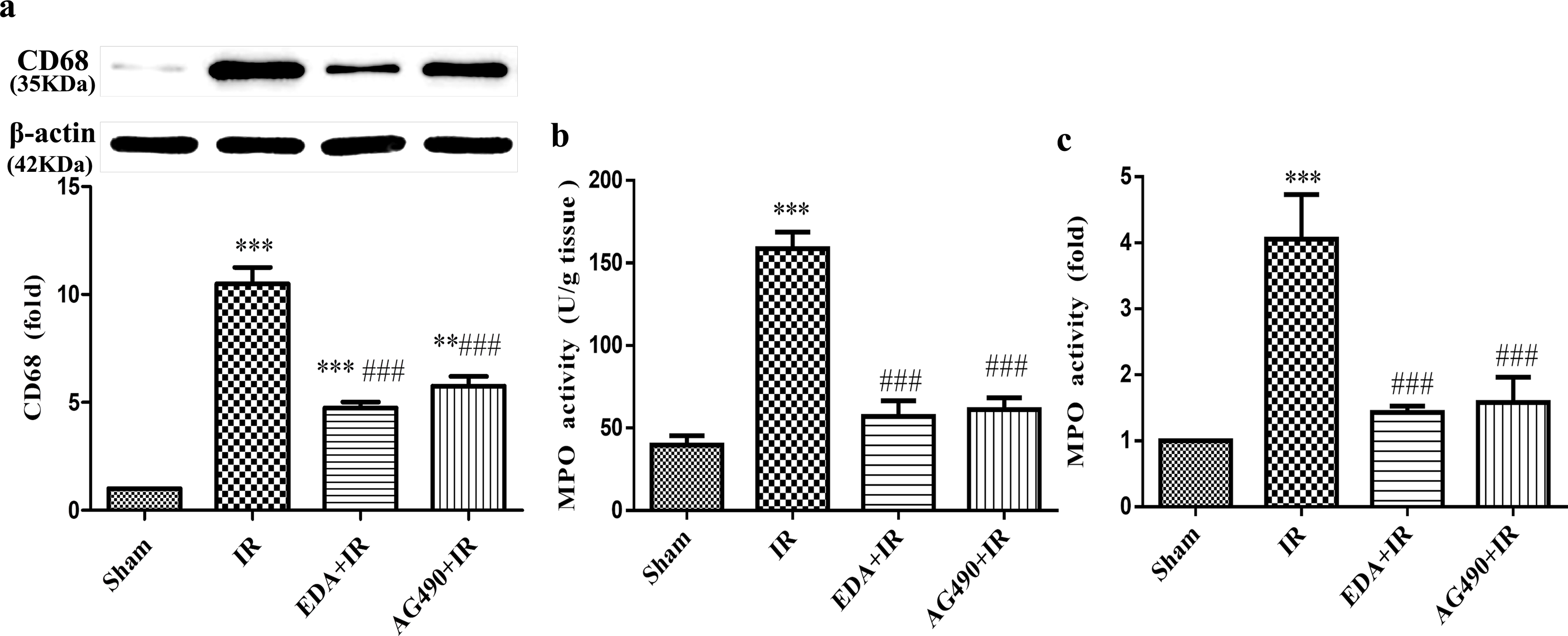

Supplement: Supplementary file 1 — Additional file 1: Figure S1. Edaravone reduces CD68 and MPO level in IR-induced kidney injury (a) The up image was the representative western blot band showed that the expression of CD68. And the below bar graph showed the expression of CD68 normalized by the sham data in each group. (b) The contents of MPO were assessed. Data are represented as mean ± SEM (n = 10 per group), ***P < 0.001 vs. sham; ###P < 0.001 vs. IR. [file 40659_2020_297_MOESM1_ESM.tif]

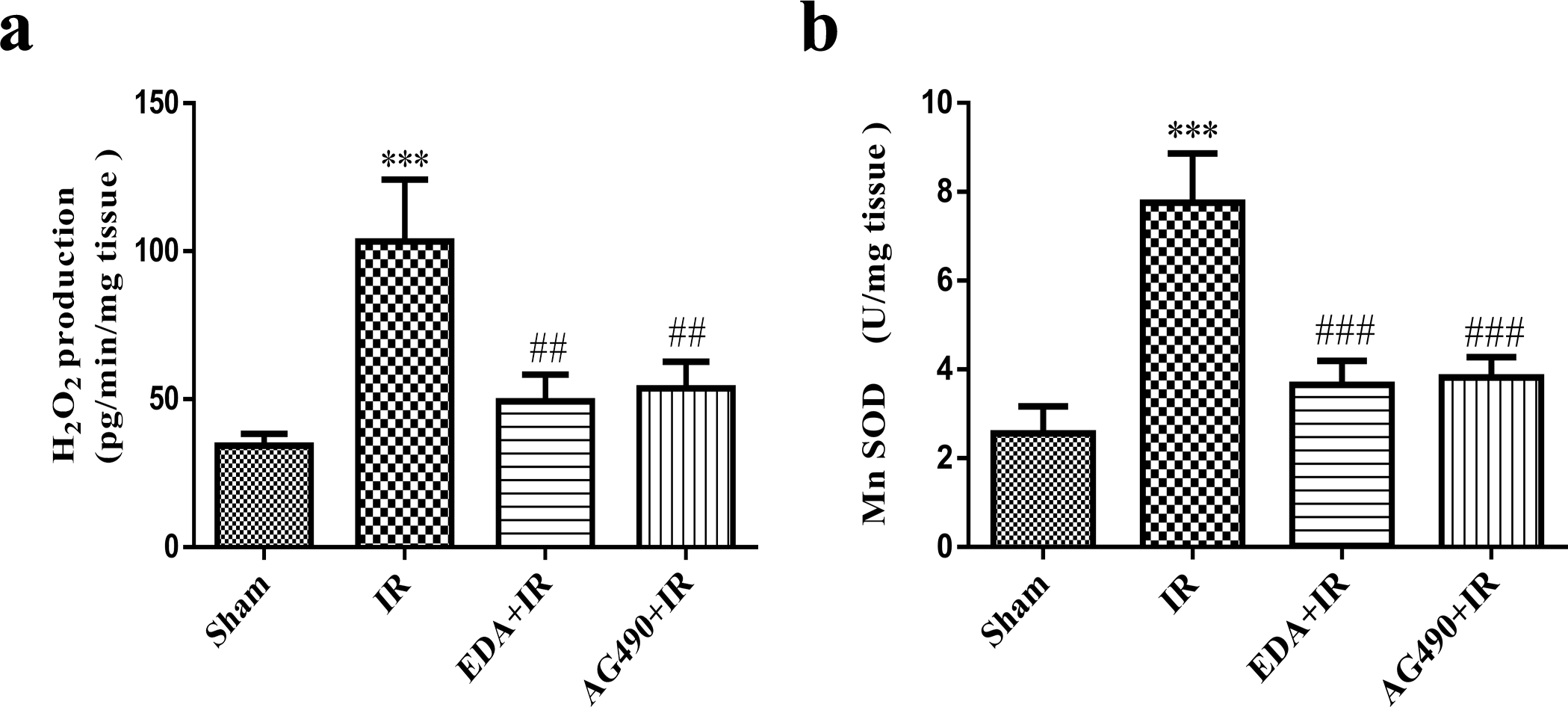

Supplement: Supplementary file 2 — Additional file 2: Figure S2. Edaravone reduces inflammatory factor production in IR-induced kidney injury. The inflammatory factors were assessed by ELISA, (a) TNF-α, (b) IL-6 and (c) IL-1β. Data are represented as mean ± SEM (n = 10 per group) *P < 0.05, ***P < 0.001 vs. sham; ###P < 0.001 vs. IR. [file 40659_2020_297_MOESM2_ESM.tif]

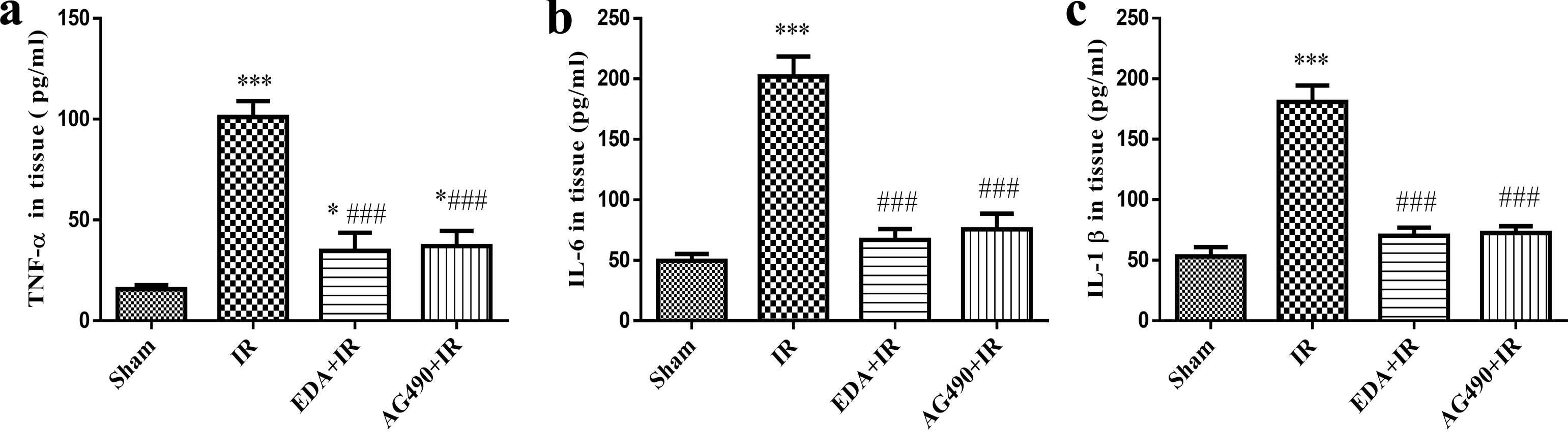

Supplement: Supplementary file 3 — Additional file 3: Figure S3. Edaravone reduces H2O2 and Mn-SOD level in IR-induced kidney injury. The H2O2 and Mn-SOD was assessed, (a) H2O2, (b) Mn-SOD. Data are represented as mean ± SEM (n = 10 per group), **P < 0.01, ***P < 0.001 vs. sham; #P < 0.05, ##P < 0.01, ###P < 0.001 vs. IR. [file 40659_2020_297_MOESM3_ESM.tif]
